# Achondroplasia: aligning mouse model with human clinical studies shows crucial importance of immediate postnatal start of the therapy

**DOI:** 10.1093/jbmr/zjae173

**Published:** 2024-10-18

**Authors:** Gustavo Rico-Llanos, Frantisek Spoutil, Eva Blahova, Adolf Koudelka, Michaela Prochazkova, Aleksandra Czyrek, Bohumil Fafilek, Jan Prochazka, Marcos Gonzalez Lopez, Jan Krivanek, Radislav Sedlacek, Deborah Krakow, Yosuke Nonaka, Yoshikazu Nakamura, Pavel Krejci

**Affiliations:** Department of Biology, Faculty of Medicine, Masaryk University, CZ-62500 Brno, Czech Republic; International Clinical Research Center, St. Anne's University Hospital, CZ-65691 Brno, Czech Republic; Czech Center for Phenogenomics, Institute of Molecular Genetics of the Czech Academy of Sciences, CZ-25250 Vestec, Czech Republic; Department of Biology, Faculty of Medicine, Masaryk University, CZ-62500 Brno, Czech Republic; International Clinical Research Center, St. Anne's University Hospital, CZ-65691 Brno, Czech Republic; Department of Biology, Faculty of Medicine, Masaryk University, CZ-62500 Brno, Czech Republic; Czech Center for Phenogenomics, Institute of Molecular Genetics of the Czech Academy of Sciences, CZ-25250 Vestec, Czech Republic; Department of Biology, Faculty of Medicine, Masaryk University, CZ-62500 Brno, Czech Republic; International Clinical Research Center, St. Anne's University Hospital, CZ-65691 Brno, Czech Republic; Department of Biology, Faculty of Medicine, Masaryk University, CZ-62500 Brno, Czech Republic; International Clinical Research Center, St. Anne's University Hospital, CZ-65691 Brno, Czech Republic; Institute of Animal Physiology and Genetics of the of the Czech Academy of Sciences, CZ-60200 Brno, Czech Republic; Czech Center for Phenogenomics, Institute of Molecular Genetics of the Czech Academy of Sciences, CZ-25250 Vestec, Czech Republic; Department of Histology and Embryology, Faculty of Medicine, Masaryk University, CZ-62500 Brno, Czech Republic; Department of Histology and Embryology, Faculty of Medicine, Masaryk University, CZ-62500 Brno, Czech Republic; Czech Center for Phenogenomics, Institute of Molecular Genetics of the Czech Academy of Sciences, CZ-25250 Vestec, Czech Republic; Department of Orthopaedic Surgery, Human Genetics, and Obstetrics and Gynecology, University of California Los Angeles, Los Angeles, CA 90095, United States; RIBOMIC Inc., Tokyo 108-0071, Japan; RIBOMIC Inc., Tokyo 108-0071, Japan; Institute of Medical Science, University of Tokyo, Tokyo 108-8639, Japan; Department of Biology, Faculty of Medicine, Masaryk University, CZ-62500 Brno, Czech Republic; International Clinical Research Center, St. Anne's University Hospital, CZ-65691 Brno, Czech Republic; Institute of Animal Physiology and Genetics of the of the Czech Academy of Sciences, CZ-60200 Brno, Czech Republic

**Keywords:** achondroplasia, Fgfr3, fibroblast growth factor, treatment, postnatal, infigratinib

## Abstract

Achondroplasia is the most common form of human dwarfism caused by mutations in the FGFR3 receptor tyrosine kinase. Current therapy begins at 2 years of age and improves longitudinal growth but does not address the cranial malformations including midface hypoplasia and foramen magnum stenosis, which lead to significant otolaryngeal and neurologic compromise. A recent clinical trial found partial restoration of cranial defects with therapy starting at 3 months of age, but results are still inconclusive. The benefits of achondroplasia therapy are therefore controversial, increasing skepticism among the medical community and patients. We used a mouse model of achondroplasia to test treatment protocols aligned with human studies. Early postnatal treatment (from day 1) was compared with late postnatal treatment (from day 4, equivalent to ~5 months in humans). Animals were treated with the FGFR3 inhibitor infigratinib and the effect on skeleton was thoroughly examined. We show that premature fusion of the skull base synchondroses occurs immediately after birth and leads to defective cranial development and foramen magnum stenosis in the mouse model to achondroplasia. This phenotype appears significantly restored by early infigratinib administration when compared with late treatment, which provides weak to no rescue. In contrast, the long bone growth is similarly improved by both early and late protocols. We provide clear evidence that immediate postnatal therapy is critical for normalization of skeletal growth in both the cranial base and long bones and the prevention of sequelae associated with achondroplasia. We also describe the limitations of early postnatal therapy, providing a paradigm-shifting argument for the development of prenatal therapy for achondroplasia.

## Introduction

Achondroplasia (ACH) is one of the most heritable forms of skeletal dysplasia and occurs in 1/15 000-40 000 live births.^1^ ACH is characterized by disproportionate short stature with an average adult height of less than 130 cm, midface hypoplasia, rhizomelic limb shortening, and vertebral pedicle shape alterations.^2^^,^^3^

ACH is caused by gain-of-function mutations in the receptor tyrosine kinase fibroblast growth factor receptor 3 (FGFR3). Most ACH patients carry the mildly activating G380R substitution in the transmembrane domain of FGFR3, which upregulates FGFR3 signaling by increasing dimerization and trans-phosphorylation within dimers.^4^ Increased FGFR3 activation affects various signaling systems required for proper chondrocyte proliferation and differentiation, including ERK MAP kinase, cytokine signaling, bone morphogenetic protein, and primary cilia/hedgehog signaling.^5–9^ Aberrant FGFR3 signaling affects chondrocyte behavior by inducing growth arrest, degradation of the cartilage extracellular matrix, cellular senescence, and dedifferentiation.^10^^,^^11^ These changes disrupt the normal architecture of growth plate cartilage and lead to impaired endochondral ossification.

At the base of the skull, bone growth occurs in synchondroses, which are cartilaginous structures consisting of two opposing growth plates joined by a common zone of resting chondrocytes. In ACH, abnormal FGFR3 signaling promotes accelerated ossification of synchondroses and premature fusion; partial or complete closure of synchondroses has been observed in prenatal or neonatal homozygous ACH.^12^ This is in contrast to the normal process of synchondrosis closure, which occurs at around 11 years of age. Premature closure leads to narrowing of the foramen magnum (FM), hydrocephalus, sudden death in infancy, and headaches in older children with ACH.^13–15^ Surgical enlargement of the small FM is indicated in 10% of children with ACH.^16^ In addition to the FM stenosis, the insufficient growth of the skull base in ACH leads to midface hypoplasia, resulting in obstructive sleep apnea, otitis media, and dental malocclusion.^17^^,^^18^

In August 2021, vosoritide was approved for the treatment of ACH.^19^^,^^20^ Vosoritide activates the C-natriuretic peptide signaling pathway, which naturally antagonizes the FGFR3 signaling pathway in growth plate cartilage.^21^ Other conceptually distinct treatments for ACH are in clinical development, such as RBM-007, an RNA aptamer that neutralizes the cognate FGFR3 ligand fibroblast growth factor 2 (FGF2), and infigratinib, a pan-specific chemical inhibitor of FGFR1-3 catalytic activity.^22^^,^^23^ Current vosoritide therapy, which begins at two years of age, increases longitudinal growth but does not treat the severe complications of ACH pathology that develop immediately or shortly after birth, such as cranial malformations and FM stenosis.^24^ This controversy fuels skepticism among patients and medical professionals, as costly vosoritide therapy does not address the serious sequelae associated with ACH, and short stature alone is not considered a diagnosis by many.^25^ A recent clinical trial found partial restoration of cranial defects with therapy starting at three months of age, but conclusions were compromised by the small numbers of participants, the limited duration of the study, and possible statistical error.^26^

The benefit of immediate postnatal treatment has yet to be clearly demonstrated; given the duration of ACH trials, it may take many years to prove it. This could divert public opinion away from ACH therapy and ultimately harm an entire generation of ACH patients who are eligible for treatment but may not receive it. In this study, we compared ACH therapy given immediately after birth with therapy started later and identified the main benefits of immediate postnatal treatment in a preclinical mouse model of ACH.

## Materials and methods

### Animal studies

All animal experiments were performed according to the guidelines of the Institutional Animal Care and Use Committee at Masaryk University, according to approved protocols (MSMT-21977/2023-3 and MSMT-6340/2021-3). Mice were maintained with ad libitum access to food and water on a regular 14 h light/10 h dark cycle, 18-23°C, and 40-60% humidity. The C57BL/6 N mice transgenic for *Fgfr3-G374R* were generated according to the procedure described before.^27^ For in vivo studies, the *Fgfr3^G374RNeoR-fl+/-^* animals were crossed with CAG-Cre^+/+^ animals, following a timed-breeding scheme.^28^ Pups were injected subcutaneously according to P1-P8, P1-P14, P4-P8, and P4-P14 protocols with 1.5 mg/kg of infigratinib (Selleckchem, Houston, TX, United States) at a volume of 10 ml/kg in buffer containing 0.9% NaCl, 3.5 mM HCl, and 200 nM cyclodextrin. Individual litters were randomly assigned into the experimental and control groups, without any distinction between males and females. At the established time points, pups were euthanized by prolonged CO_2_ inhalation.

### Histology, vital dyes, hard tissue clearing, and histology

Bone vital staining and hard tissue clearing were performed following a protocol previously described, with slight modifications.^29^ Briefly, pups were injected intraperitoneally with 6 mg/kg of calcein (Sigma-Aldrich, MA, United States) 18-24 h before euthanasia. Animals for day 8-analyses were additionally injected with 30 mg/kg of alizarin red S complex (Sigma-Aldrich) at P3. Both compounds were dissolved in a 2% NaHCO_3_ solution in sterile conditions. Skulls were dissected, washed, and cleared as described before.^29^ P14 mice were pictured in supine and lateral positions. Then, they were weighed, and naso-anal length was measured with a digital caliper. For histology, skulls, tibias, and femurs were decalcified in Q Path Decalcifier DC1 (VWR, PA, United States) for 4-8 days, dehydrated, embedded in paraffin, sectioned at 8 μm, and stained following a standard alcian blue, hematoxylin, and eosin protocol.

### Micro-computed tomography and image analysis

For micro-computed tomography (μCT) imaging, animals were externally washed in PBS and fixed without any tissue removal or dissection by immersion in buffered 4% formaldehyde for two weeks at 4 °C. Before scanning, the whole animals were dried with a paper towel and embedded in 2.5% low melting agarose (Sigma-Aldrich) in 50 ml plastic tubes. Scan features and descriptions of the region of interest (ROI) definitions are reported according to the established guidelines for rodent μCT analysis.^30^ Animals were scanned in Bruker SkyScan 1176 (Bruker, MA, United States) on the resolution of 8.65 μm and full rotation scan with step of 0.33°; 1 mm aluminum filter was used with X-ray source current of 370 μA and voltage of 50 kV. Two-times averaging with an exposition of 2 s was used per position of the X-ray source. Virtual sections were reconstructed in NRecon 2.2.0.6 (Bruker) with smoothing set up to 4, ring artifact correction to 11, beam hardening correction to 28%, and defect pixel mask to 5%. The range of attenuation units for reconstruction was set from 0.007 to 0.150. CT-Vox 3.3.0 (Bruker) was used to orient 3D datasets and produce 2D captures of skulls, femurs, and tibias. Lumbar vertebrae length (L4-L6) was defined as the distance between the most caudal point of the vertebral body in L6 to the most rostral point of the vertebral body in L4 from a ventral view. Femur length was defined as the distance between the greater trochanter and the distal growth plate from a rostral view, whereas tibia length was measured from the medial malleolus to the proximal growth plate from a rostro-lateral view. Cortical bone density is defined as the mean pixel gray intensity value (in the 2D capture produced from the 3D reconstruction) within the whole ROI defined as the femur diaphysis. Skull length was measured from a dorsal view as the distance between the most anterior point of the nasal bone and the most posterior point of the occipital bone. The FM area was measured from an inferior parallel transversal plane. Representative schemes of how these measurements were done are shown in Figure S4. Other 2D captures were also produced for nonquantitative observations like an internal dorsal view of the floor of the cranial cavity (for synchondroses visualization) and a lateral view of the skull. Measurements were performed in ImageJ 1.54.

### Statistical analyses

The *n* values express the actual number of mice stated in each figure panel and summarized in Table S1. In graphs, the dots represent individual animals, and the lines and whiskers represent mean ± SD. Two-way ANOVA with post hoc Tukey multiple comparisons test was used for statistical evaluation. All statistical analyses were performed using GraphPad Prism version 8.0.2.

## Results

**Figure 1:**
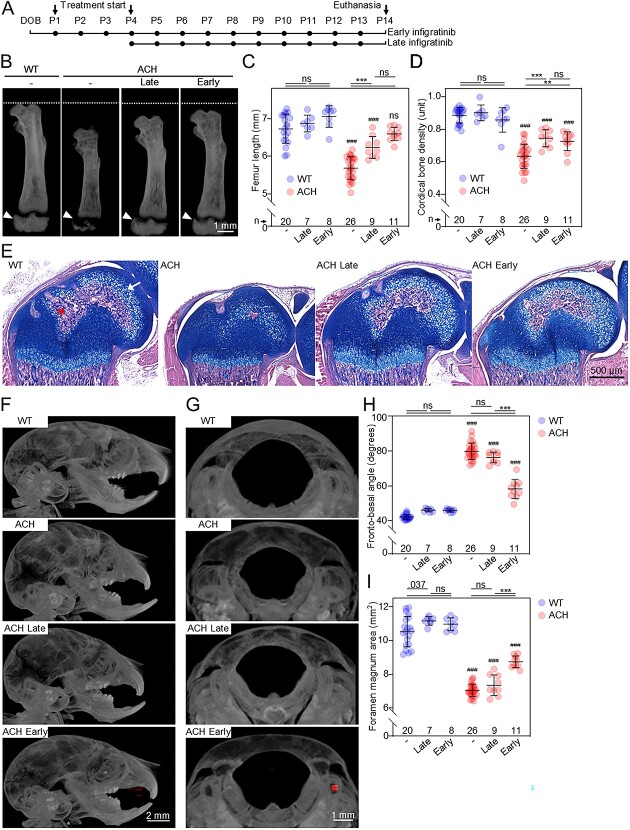
Early treatment with infigratinib rescues foramen magnum stenosis in ACH mice. (A) Schematic representation of infigratinib protocols. (B) Representative images of femurs with indicated length of untreated wildtype (WT) mice (dashed line). The arrows indicate secondary ossification centers in the distal part of the femur. (C, D) Skeletal phenotypes of ACH mice were determined by image analysis of μCT-3D reconstructions and graphed. Measurements of (C) femur length and (D) femur cortical density. Points; individual animals; lines and whiskers, mean ± SD; n, number of animals; statistically significant differences are indicated, ^**^p<.01, ^***^p<.001; #, comparison with corresponding WT animals. (E) Sections of distal femoral epiphyses, stained with hematoxylin/eosin (bone and soft tissues) and alcian blue (cartilage) (arrow, hypertrophic cartilage; arrowhead, bone). Poorly developed secondary ossifications in ACH femurs were corrected by both early and late infigratinib treatments. (F, G) Representative images lateral view of the skull, and foramen magnum. The fronto-basal angle (Figure S4) (H) and foramen magnum area (I) were measured and graphed. Increased fronto-basal angle and smaller foramen magnum in the ACH animals were corrected by early but not by late infigratinib treatment.

The inhibitor of FGFR catalytic activity infigratinib (BGJ398) was selected for animal experiments because it is currently being investigated in ACH clinical trials,^23^ and shows efficacy in the preliminary studies carried out on the *Fgfr3^G374R/wt^* C57BL/6 N mice used here to model ACH (Figure S1). The G374R mouse mutation is orthologous to human G380R mutation, which accounts for 99% of ACH cases.^31^ The *Fgfr3^G374R/wt^* mice faithfully recapitulate pathological effects of aberrant FGFR3 on skeletal growth observed in humans.^27^ Published growth curves of C57BL/6 N mice show a growth spurt in the first 3 postnatal weeks before weaning at postnatal day 21 (P21).^32^ In humans, the period of initial intensive growth ends around 2 years when it is replaced by more graduate linear growth. Assuming the general correlation between mouse and human age (1 mouse day to 40 human days),^33^ the administration of vosoritide to ACH patients aged 2 years corresponds to ~P18 in mice. We chose the P1-P14 treatment protocol (referred to as the “early postnatal”) instead of P1-P21 protocol, in order to reduce in vivo experimentation and animal suffering while still covering ~70% of the preweaning growth spurt. A protocol that began at P4 (~5 postnatal months in humans) was used as an alternative to the early protocol. The P4-P14 protocol is referred to as “late postnatal” (Figure 1A). Infigratinib was chosen over vosoritide because it is currently being tested in clinical trials for the treatment of achondroplasia, has shown robust effects in achondroplasia mouse models, and is significantly less expensive than the commercially available vosoritide.^23^^,^^34^ Infigratinib was administered by once-daily subcutaneous injection of 1.5 mg/kg (Figure S1), and skeletal tissues were examined by μCT at P14.

**Figure 2:**
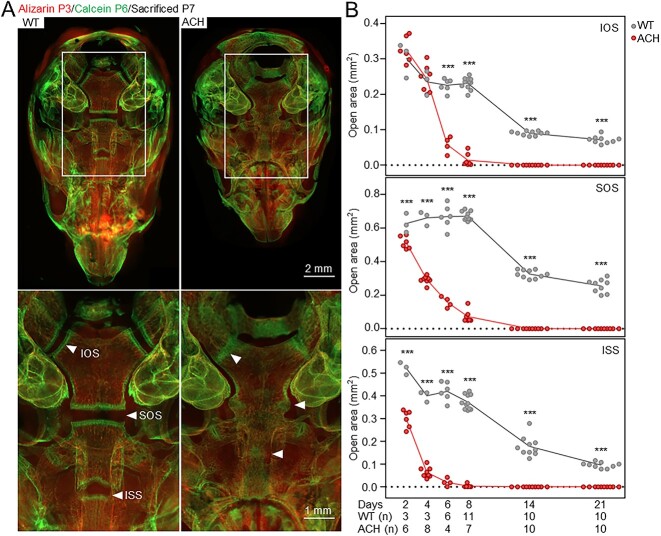
Premature synchondroses fusion in ACH mice. (A) Representative images of cranial basis of WT and ACH mice at P7, visualized by intravital staining with alizarin red at P3 and calcein at P6. Intraoccipital anterior (IOS), sphenoid occipital (SOS), and intersphenoidal (ISS) synchondroses are indicated (arrowheads). Note the complete IOS, SOS, and IOS fusion in ACH mice at P7, caused by the replacement of the growth plate cartilage element (visible as a black gap in images) by bone. (B) Dynamics of the synchondroses closure, generated by measurements of cartilage tissue area of IOS, SOS, and ISS between P2 and P21 (Figure S5). Synchondroses were visualized by intravital calcein staining, and documented by fluorescence microscopy (P2-P8) or by μCT images (P14-P21); cartilage areas were determined by image analysis of the dark zone between the two edges of every synchondrosis. For IOS, the values correspond to the average between the left and the right IOS. The numbers of animals are indicated (n). Points; individual animals; statistically significant differences between WT and ACH are indicated (^***^p < 0.001).

**Figure 3:**
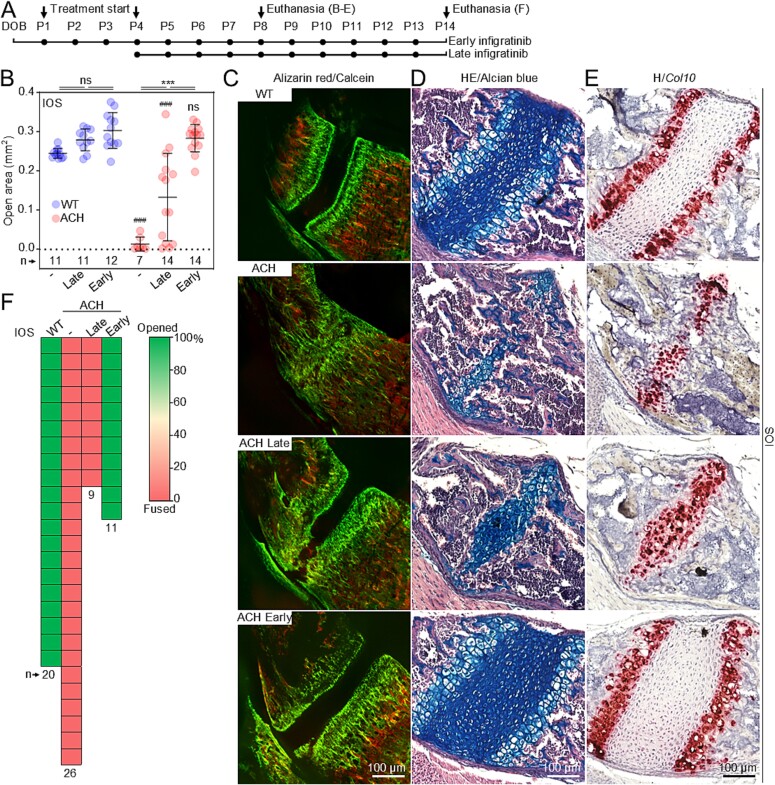
Early treatment with infigratinib rescues premature fusion of IOS. (A) Schematic representation of infigratinib protocols. (B) Quantification of the IOS open area in response to late and early infigratinib treatment at P8. Points; individual animals; lines and whiskers, mean ± SD; n, number of animals; statistically significant differences are indicated, ^***^p < 0.001; #, comparison with corresponding wildtype (WT) animals; ns, not significant. (C) Representative images of the IOS open areas for late and early infigratinib treatments compared with non-treated WT mice after intravital alizarin red (P3) and calcein (P7) staining. (D) Corresponding histological sections stained with alcian blue/HE, to visualize growth plate cartilage (blue). (E) RNAscope in situ hybridization for *collagen 10* expression. (F) The extent of IOS fusion at P14 (representative images in Figure S6); each box represents one animal.

### Infigratinib improves defective axial and appendicular growth in ACH mice

The impaired axial skeleton growth typical of human ACH is recapitulated in ACH mice, as their body length at P14 was 23.1 ± 4% (*n* = 26) shorter than in wildtype animals (Figure S2A). The early treatment led to significant rescue of the naso-anal length in ACH animals, which were 18.6 ± 5.1% (*n* = 10) longer when compared with untreated ACH animals (Figure S2A). This was consistent with the observed rescue in vertebral growth, as demonstrated by measurements of lumbar vertebrae (L4-L6) and the tail length. We observed 14.8 ± 6% (*n* = 10) increase in L4-L6 length and 18.4 ± 8.9% (*n* = 10) in tail-length, respectively, in animals treated with early protocol when compared with the untreated ACH animals (Figure S2B and C). Late infigratinib treatment failed to rescue the naso-anal length, L4-L6 length and tail length.

Regarding the appendicular skeleton, measurements of the femur length from reconstructed μCT scans revealed impaired growth by 15.6 ± 4.4%. This was restored with both infigratinib protocols, as the ACH femurs were only 2.0 ± 2.5% (early) and 7.3 ± 4.1% (late) shorter than in wildtype littermates (Figure 1C). Similar rescue was found in the tibias, which were growth-impaired by 20.6 ± 4.1% in ACH mice and restored to 6.7 ± 2.1% and 13.1 ± 4.1% of wildtype mice by early and late infigratinib protocols, respectively (Figure S3). No significant differences were found between late and early infigratinib protocols in the femurs (*p* = 0.129), in contrast to tibias, where early treatment produced a significantly bigger effect when compared with the late treatment (*p* = 0.022). This is likely due to temporal differences in growth of individual skeletal elements which may determine their response to the therapy.

Cortical bone density analysis generated by 3D μCT imaging showed a substantial correction in the femoral diaphysis of ACH animals treated with both protocols (Figure 1D). We also noted poorly developed secondary ossification centers in ACH femurs and tibias, suggesting delayed ossification (Figs 1B and S3; arrows). Femur histology revealed well-developed secondary ossifications containing fully differentiated hypertrophic chondrocytes and primary bone in infigratinib-treated animals, whereas no bone tissue was found in untreated ACH femurs at P14 (Figure 1E). This phenotype was also restored by both infigratinib protocols.

### Early infigratinib treatment restores defective head skeletogenesis in ACH mice

The general skull morphology of the untreated ACH mice was consistent with the changes seen in human ACH that include midface hypoplasia, frontal bossing, maxillary hypoplasia, and malocclusion due to insufficient growth of the skull base^18^ (Figure 1F). For simplicity, the skull shape was characterized by the fronto-basal angle formed between the sella turcica, the nasal bone, and the most anterior point of the frontal bone in the mid-sagittal plane of the lateral view (Figure S4). The fronto-basal angle was dramatically increased in ACH animals compared with wildtype littermates (79.8 ± 4.75^o^ in ACH, *n* = 26 vs. 42.1 ± 1.46^o^ in wildtype, *n* = 20). Early treatment with infigratinib partially normalized the fronto-basal angle, bringing it down to 58.2^o^ ± 5.48^o^ (*n* = 10). In contrast, late treatment did not improve the fronto-basal angle (76.3^o^ ± 3.06^o^, *n* = 9) (Figure 1H). Next, the FM was examined (Figure 1G). Measurements of the FM area revealed noteworthy stenosis in ACH animals (7.0 ± 0.36 mm^2^ in ACH vs. 10.5 ± 0.90 mm^2^ in wildtype; *p*<0.001), with no obvious changes in FM shape. Similar to fronto-basal angle, a significant recovery of FM area was observed with the early infigratinib protocol, whereas no rescue was observed in late-treated animals (Figure 1I). Taken together, our data show that both early and late infigratinib protocols induce recovery of impaired appendicular skeletal growth in ACH mice, with a stronger effect of the early protocol on tibia length. However, only early infigratinib treatment restores axial skeleton growth and defective cranial base skeletogenesis, determined by the measurements of the fronto-basal angle and the FM area.

Next, the mechanism underlying the infigratinib effect on skull morphology was investigated. Morphologic examination of the skull base of ACH mice revealed premature fusion of the intraoccipital (IOS), sphenoccipital (SOS), and intersphenoidal (ISS) synchondroses at P7, in contrast to wildtype littermates, which showed no evidence of closure (Figure 2A). Analysis of the postnatal dynamics of the IOS, SOS, and ISS in ACH mice (Figure S5) revealed that IOS does not close until ~P5, whereas SOS begins to close as early as P2. Interestingly, ISS appeared to be approximately 43% closed by P2, suggesting that premature ISS closure associated with ACH begins in the prenatal period (Figure 2B).

**Figure 4:**
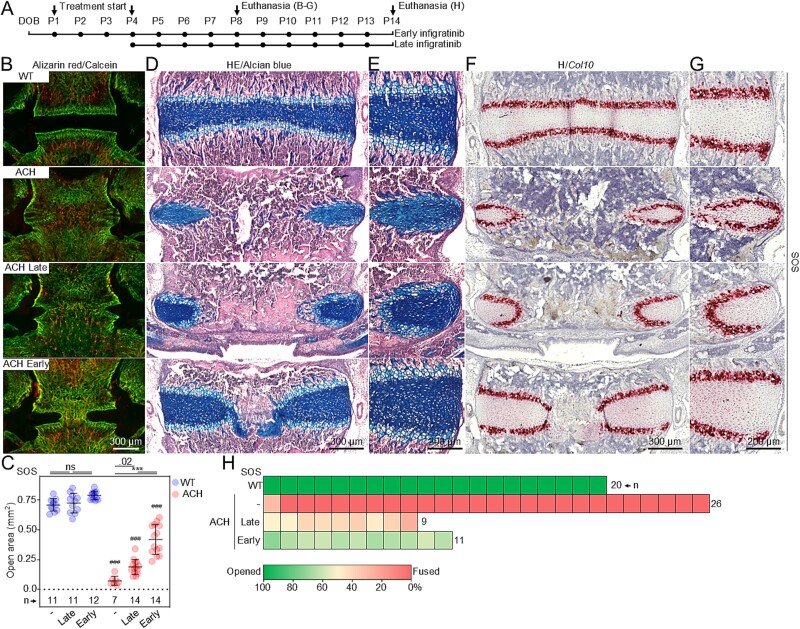
Early treatment with infigratinib rescues premature fusion of SOS. (A) Schematic representation of infigratinib protocols. (B) Representative images of the SOS open areas for late and early infigratinib treatments compared with non-treated wildtype (WT) mice after intravital alizarin red (P3) and calcein (P7) staining. (C) Quantification of the SOS open area in response to late and early infigratinib treatment at P8. Points; individual animals; lines and whiskers, mean ± SD; n, number of animals; statistically significant differences are indicated, ^***^p < 0.001; #, comparison with corresponding WT animals; ns, not significant. (D, E) Corresponding histological sections stained with alcian blue/HE to visualize growth plate cartilage (blue). (F, G) RNAscope in situ hybridization for *collagen 10* expression. (H) The extent of SOS fusion at P14 (representative images are shown in Figure S7); each box represents one animal.

Early postnatal infigratinib protocol resulted in significant rescue of the premature fusion of IOS at P8 (Figure 3A-C). Specifically, quantification of the cartilaginous tissue areas within the synchondroses showed 93.5 ± 11.1% (*n* = 14) rescue in IOS (100%, fully open in wildtype; 0%, fully closed in ACH). In contrast, the late postnatal protocol had a significantly weaker (*p*<.001), and highly heterogeneous effect, achieving only 47.7 ± 38.4% of salvage of the IOS cartilage at P8. Even though for SOS both protocols delayed the premature fusion to some extent, early infigratinib treatments produced 53.1 ± 15.3% of rescue of the synchondrosis cartilage, compared with 26.0 ± 8.3% evidenced for the late protocol (Figure 4A-C). The histology of the IOS in ACH mice treated with early infigratinib protocol resembled their wildtype littermates, with 2 opposing growth plate cartilage zones containing resting, proliferating, and well-differentiated hypertrophic chondrocytes (Figure 3D). This was confirmed by RNAScope analysis of *collagen type 10* expression, a marker for hypertrophic chondrocytes (Figure 3E). In contrast, ACH animals treated with late infigratinib showed a disorganized hypertrophic zone in the final stages of the endochondral ossification process, eventually leading to complete fusion. In SOS, accelerated endochondral ossification spread from the center and led to the formation of a bony bridge. Early, but not late, treatment with infigratinib delayed this process (Figure 4D-G). The above observations demonstrate the importance of early intervention to ameliorate premature fusion of the synchondroses. Remarkably, only in the animals treated early with infigratinib the IOS were still open even at P14 (Figures 3F and S6). Consistently with what was observed at P8, even though both protocols improve the premature fusion the SOS, the cartilage in the early treatment covered a substantially higher proportion of the basisphenoid bone (17% late vs. 51.9% early infigratinib) (Figures 4H and S6).

**Figure 5:**
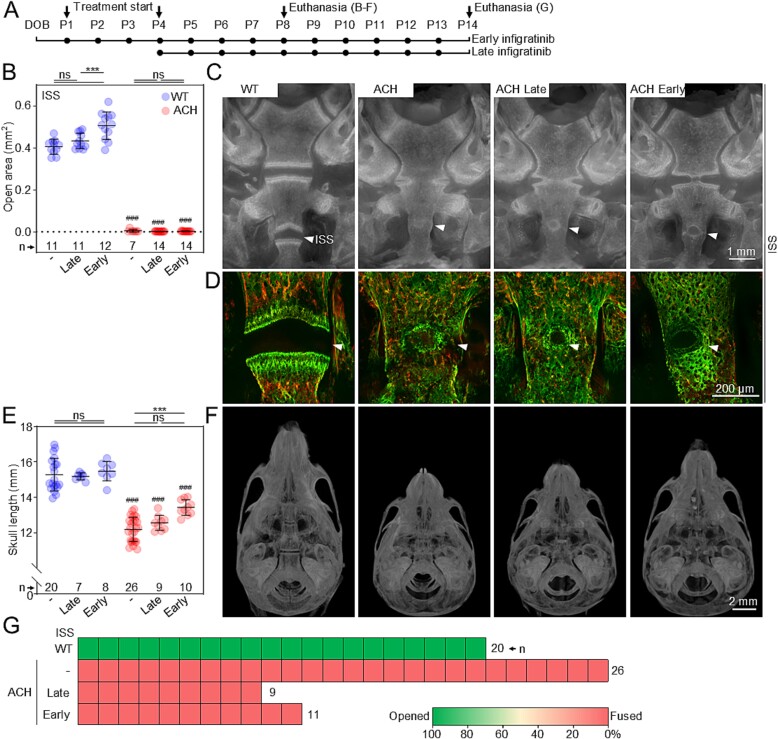
No infigratinib effect on premature ISS fusion in ACH. (A) Schematic representation of infigratinib protocols. (B) Quantification of the ISS open area in response to late and early infigratinib protocol at P8. Graph points; individual animals; lines and whiskers, mean ± SD; n, number of animals; statistically significant differences are indicated (^***^p < 0.001). (C, D) Representative images of ISS in wildtype (WT) and ACH animals treated with early (P1-P8) and late (P4-P8) infigratinib protocols. The bone was visualized by intravital alizarin red (P3) and calcein (P7) staining. (E) Changes in skull length in mice treated with late and early infigratinib protocols up to P14. (F) Representative μCT images from a dorsal view of the skull. (G) The extent of ISS fusion at P14 (representative images are shown in Figure S7); each box represents one animal.

Interestingly, our results showed that, regardless of the treatment protocol, no effect of infigratinib on ISS was observed (Figure 5A-D and G). Since ISS contributes substantially to the growth of the skull base of mice along the rostro-caudal direction, a significant (*p* < 0.001) but relatively weak effect of infigratinib on ACH skull length was observed (Figure 5E-F).

## Discussion

In this study, we show that synchondroses closures progress rapidly in the immediate postnatal period of ACH mice and are completed shortly after birth at P8 (equivalent to 11 months in humans). Similarly, rapid premature closures are also observed in ACH patients, with the IOS closing between 7 and 24 months and the SOS closing between 16 months and 5 years.^35–37^ This dynamic implies that ACH therapies starting at 2-4 years of age are unlikely to be successful in preventing cranial malformations and FM stenosis. Although longitudinal growth (stature height) will be improved, these ACH patients may continue to suffer from severe and lifelong neurological complications. We provide clear experimental evidence for this assumption by showing that while both early and late postnatal treatments increase long bone growth in ACH mice, only early treatment results in significant improvement of defective cranial base skeletogenesis. Therefore, ACH patients should be treated immediately after birth to maintain synchondrosis growth and prevent neurological complications.

Alarmingly, even if treatment is started immediately after birth, it cannot restore some of the skeletal abnormalities like the ISS premature closure. We found no human data on detailed postnatal ISS dynamics in ACH, and there are likely differences in normal ISS growth between the two species subject to ecological olfactory adaptation (microsmatic mouse vs. microsmatic human). In some primates, such as lemurs and monkeys, the ISS is fully open at birth and shows two clear opposite cartilage growth plates. In primates more closely related to humans, such as chimpanzees, the ISS is no longer clearly visible as early as 5 days after birth, suggesting a close relationship between ISS fusion dynamics and midface projection.^38^^,^^39^ Few studies address this feature in humans, where the ISS shows bony bridging across the edges as early as 1 month after birth and complete fusion after 2 to 3 years.^40^ In ACH patients, the ISS appears to start fusing during embryonic development, and complete fusion occurs more rapidly. A multimodal study conducted in 15 pediatric ACH patients showed that 11 of them had premature fusion of the ISS compared with control patients.^37^ Frontal bossing and midface hypoplasia are often seen on prenatal ultrasound in achondroplasia starting in the early third trimester (Figure S7), suggesting that the ISS fusion process also begins before birth in ACH patients. Why aberrant FGFR3 activation leads to a rapid decline in ISS, as opposed to more gradual effects on SOS and IOS, remains to be investigated. Future research should also uncover the molecular and cellular processes that precede the onset of fusion and the reasons why the ongoing fusion process cannot be reversed by FGFR3 inhibition.

Overall, we show that some cranio-skeletal defects associated with ACH cannot be successfully treated after birth, clearly emphasizing the need for the development of prenatal therapy for ACH. This is not an unbroken ground, as prenatal therapy was recently shown to correct X-linked hypohidrotic ectodermal dysplasia (XLHED), a rare genetic disorder caused by a lack of sweat gland development due to a deficiency in ectodysplasin A (EDA). Two injections of the recombinant receptor-binding domain of EDA fused to an Fc fragment of immunoglobulin into the amniotic fluid, performed at gestational weeks 26 and 31, restored sweat gland development, resulting in normal perspiration after birth.^41^

## Supplementary Material

Supplementary_Material_JBMR_final_zjae173

Supplementary_Table_1_zjae173

## Data Availability

The data that support the findings of this study are available from the corresponding author upon reasonable request.
