# Supplementary material for: Achondroplasia: aligning mouse model with human clinical studies shows crucial importance of immediate postnatal start of the therapy
Source: J Bone Miner Res. 2024 Oct 18;39(12):1783–92. doi: 10.1093/jbmr/zjae173 (PMC11638852; doi:10.1093/jbmr/zjae173)
Supplement: Supplementary_Material_JBMR_final_zjae173 [file supplementary_material_jbmr_final_zjae173.docx]

**Supplementary Figures**

**Figure S1** Evaluation of infigratinib toxicity and efficacy


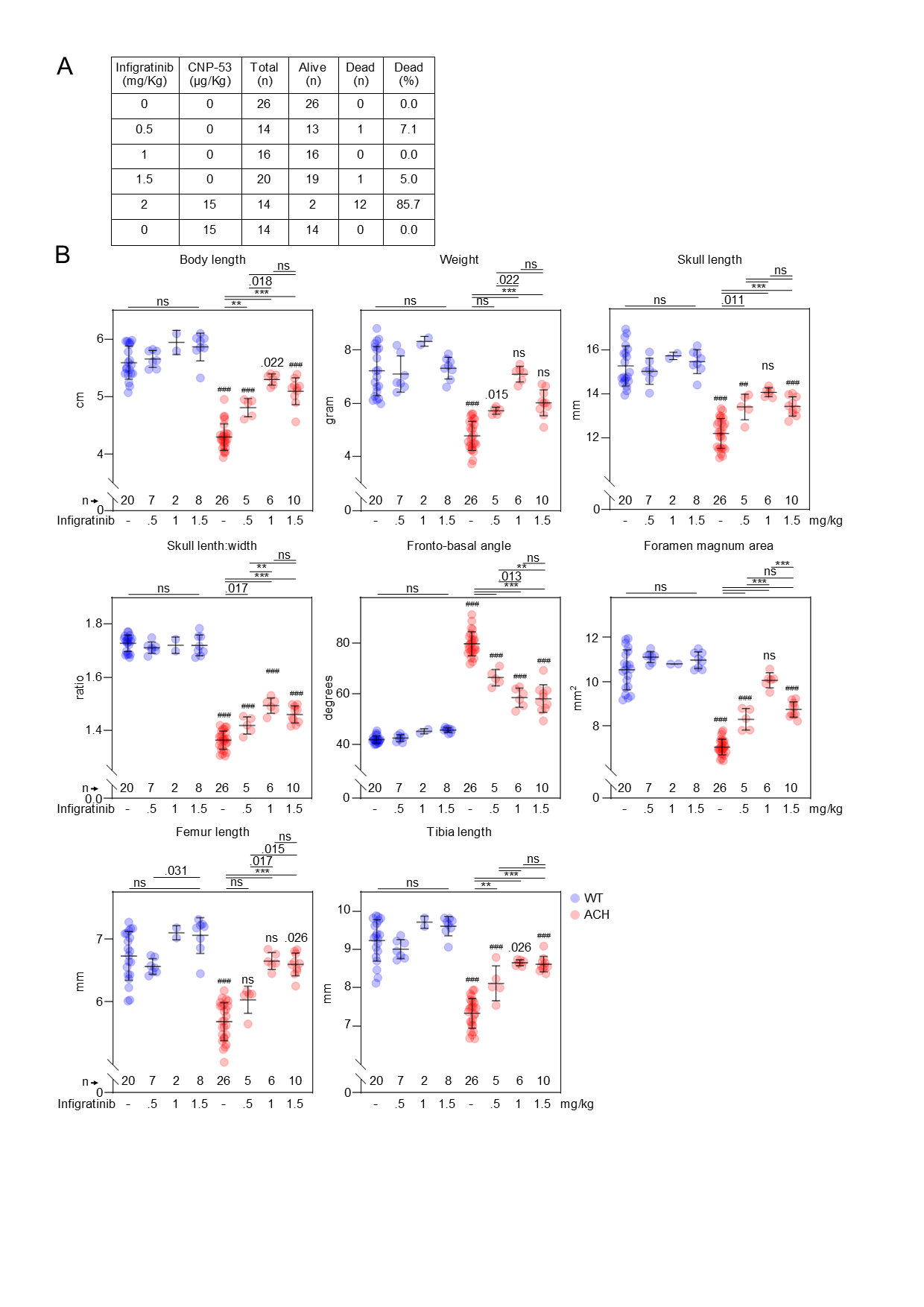


(**A**) Survival rates for mice treated with different doses of infigratinib in the P1-P14 protocol; 2 mg/kg of infigratinib combined with an innocuous dose of C-natriuretic peptide 53 (CNP53) (15 µg/kg) shows a high mortality rate. (**B**) Infigratinib effect on body length, weight, skull length, skull length/width ratio, fronto-basal angle, foramen magnum area, and femur and tibia length. Pictures were obtained from WT and ACH mice treated with 0.5, 1 and 1.5 mg/kg of infigratinib in the P1-P14 protocol and compared with non-treated mice as controls. Points; individual animals; lines and whiskers, mean±SD; n, number of animals; statistically significant differences are indicated, **p<0.01, ***p<0.001; #, comparison with corresponding WT animals; ns, not significant.

**Figure S2** Infigratinib effect on axial skeletal growth in ACH mice


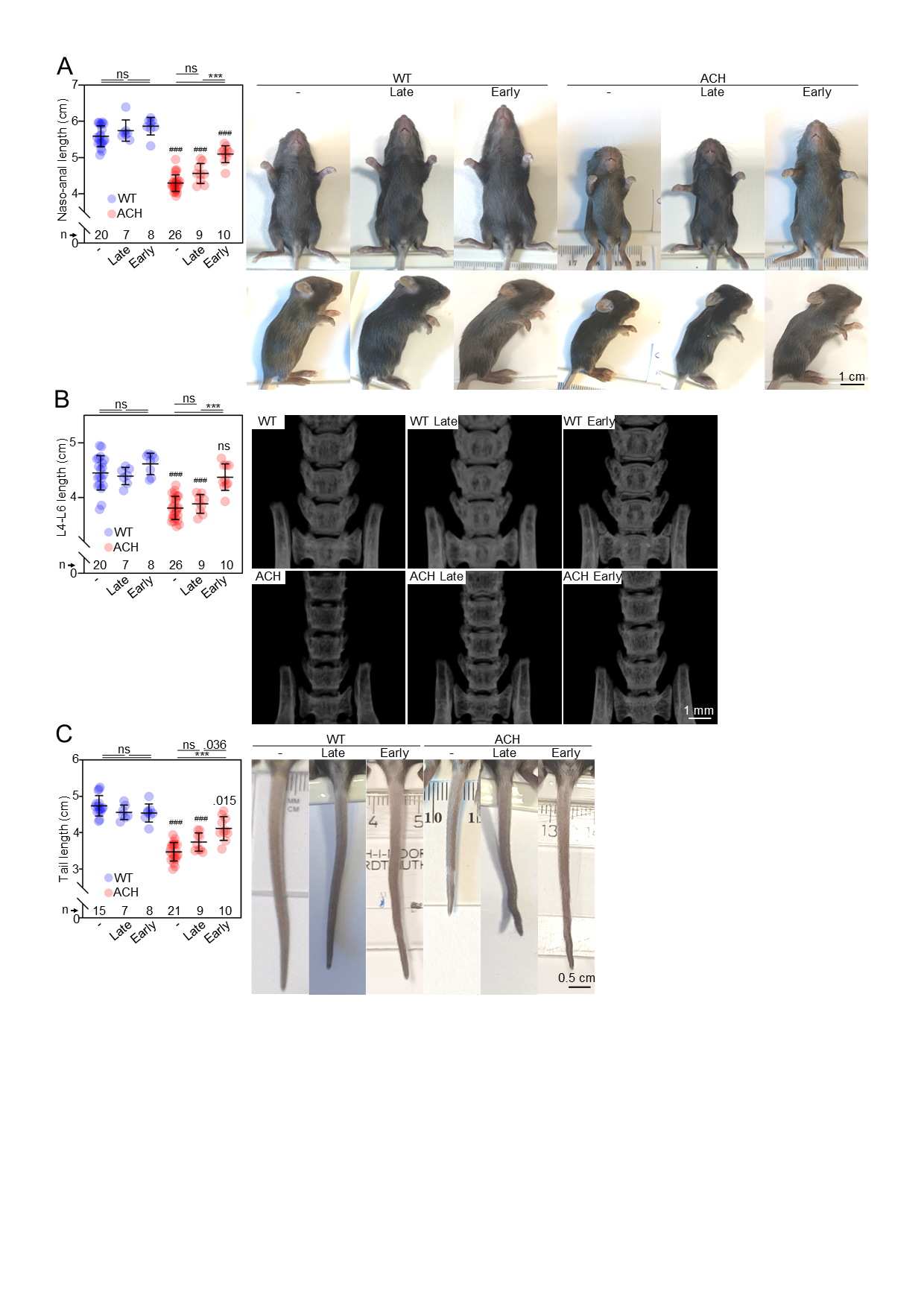


(**A**) Naso-anal length, (**B**) lumbar vertebrae L4-L6 length and (**C**) tail length measurements of the ACH and wildtype (WT) mice treated with early (P1-P14) and late (P4-P14) infigratinib protocols. Points; individual animals; lines and whiskers, mean±SD; n, number of animals; statistically significant differences are indicated, ***p<0.001; #, comparison with corresponding WT animals; ns, not significant.

**Figure S3** Infigratinib effect on tibia growth in ACH mice


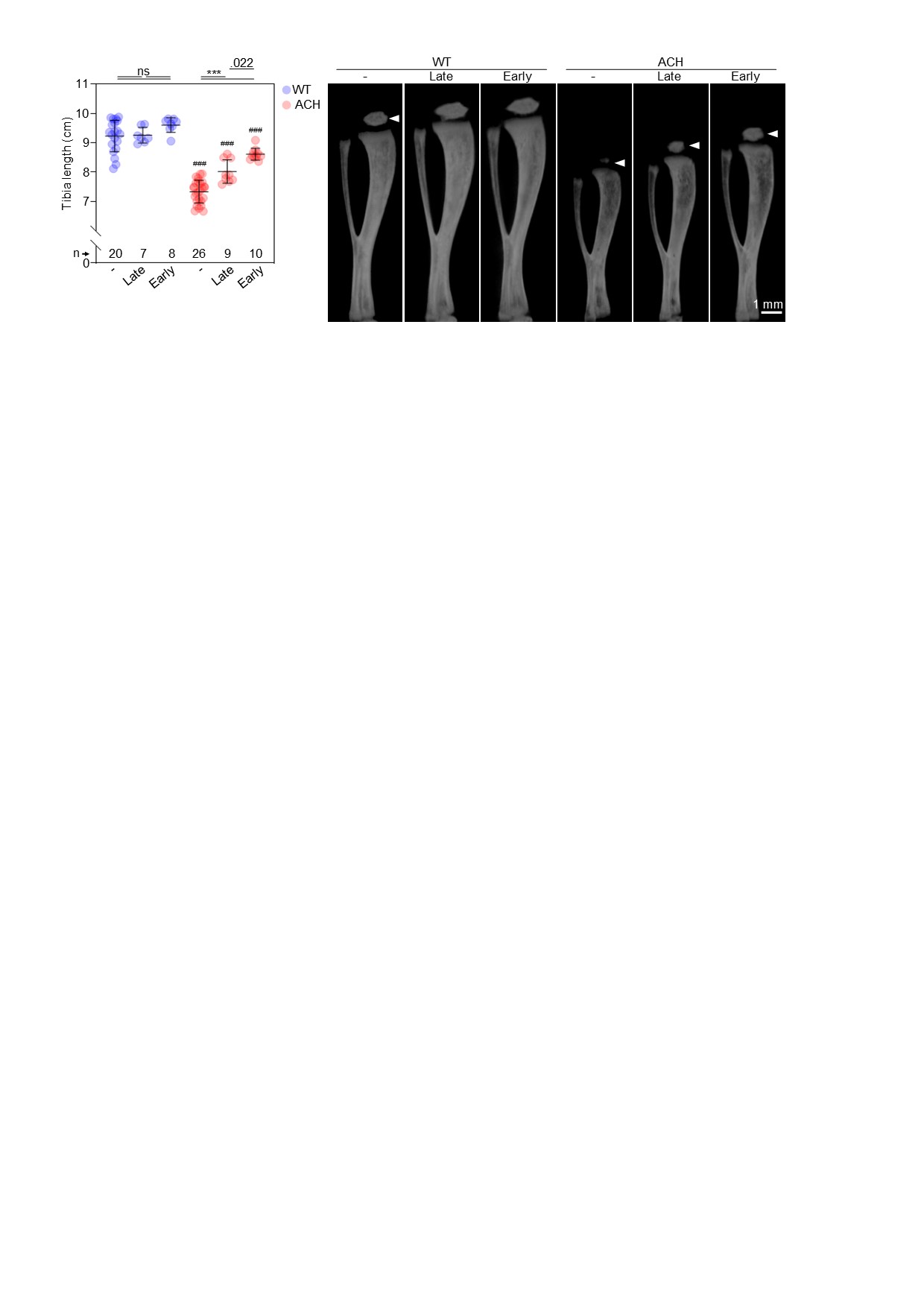


General appearance of tibia isolated from ACH and wildtype (WT) mice treated with early (P1-P14) and late (P4-P14) infigratinib protocols. Arrows indicate secondary ossification centers in the proximal end of the tibia. Graph points; individual animals; lines and whiskers, mean±SD; n, number of animals; statistically significant differences are indicated, ***p<0.001; #, comparison with corresponding WT animals.

**Figure S4** Schematic representation of the fronto-basal angle


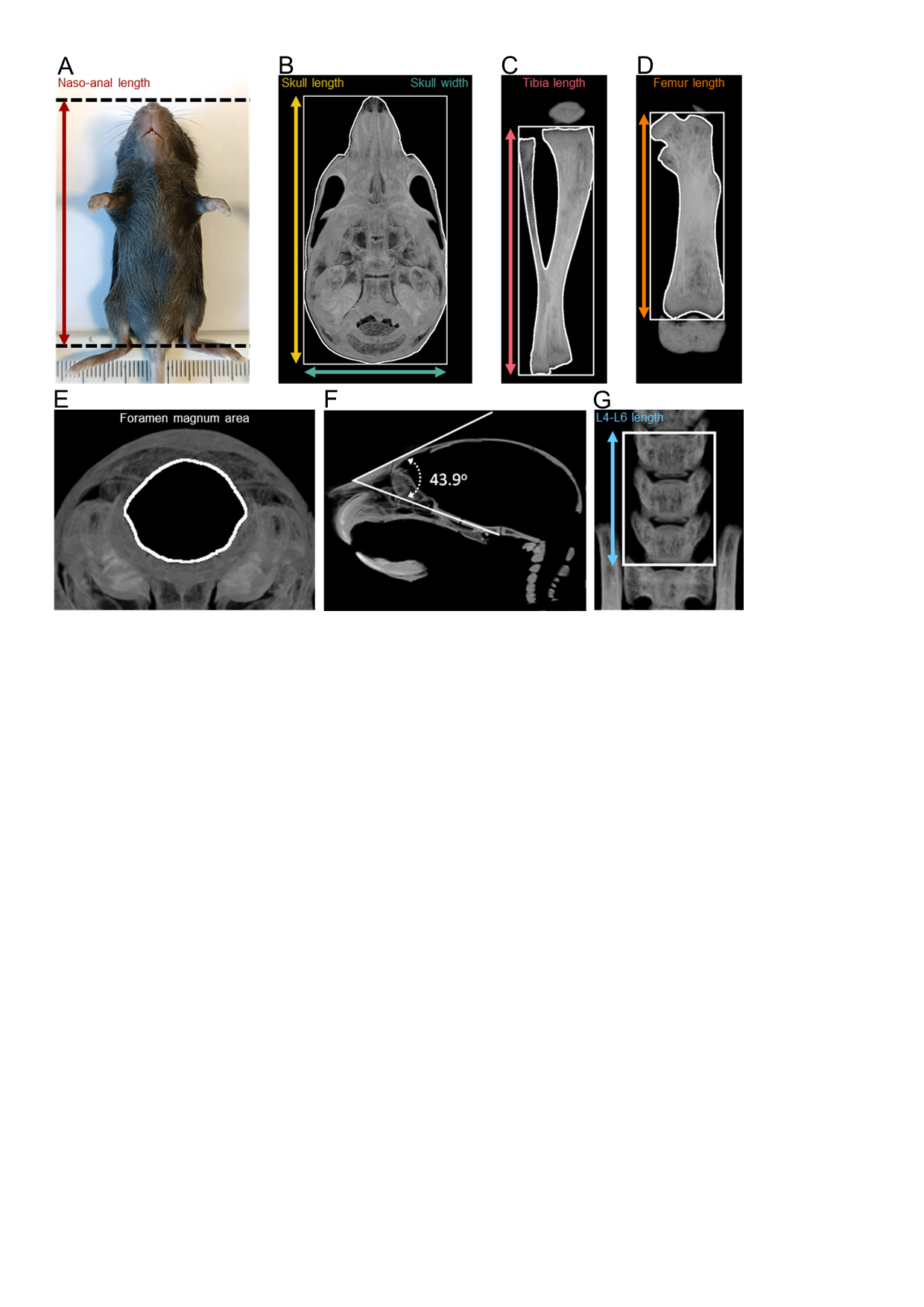


Examples of the selected regions of interest (ROIs) used to generate the measurements of (**A**) body length, (**B**) skull length and width, (**C**) tibia and (**D**) femur length, (**E**) foramen magnum, (**F**) fronto-basal angle and (**G**) L4-L6 length.

**Figure S5** Analysis of the postnatal dynamics of IOS, SOS and ISS


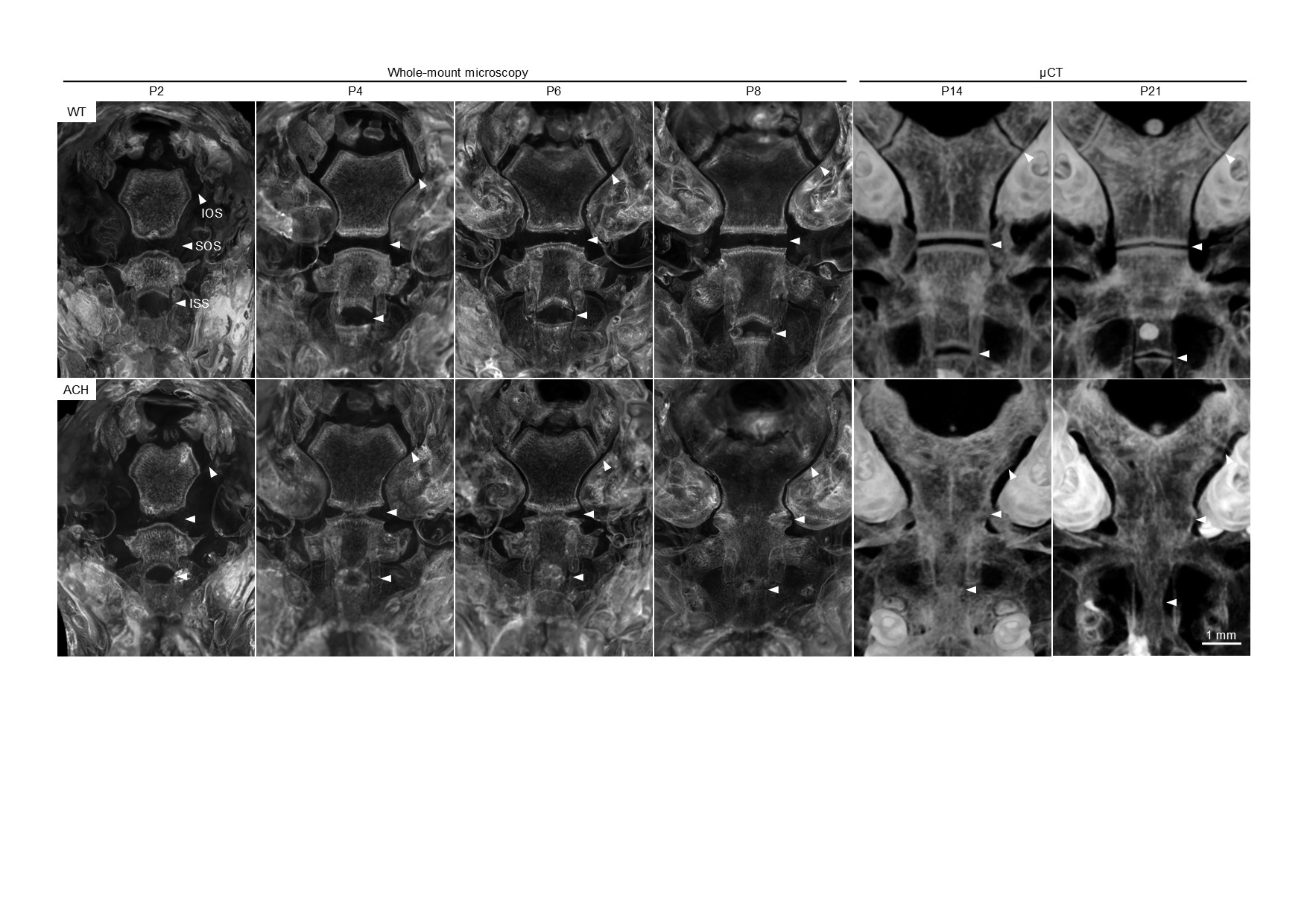


Representative images of whole-mount fluorescent preparations (P2-P8) and reconstructed microCT scans (P14, P21) were used to measure the extent of synchondrose closure (arrows). The areas were measured by image analysis as the black space between the two edges of the synchondroses.

**Figure S6** The extent of synchondroses fusion at P14


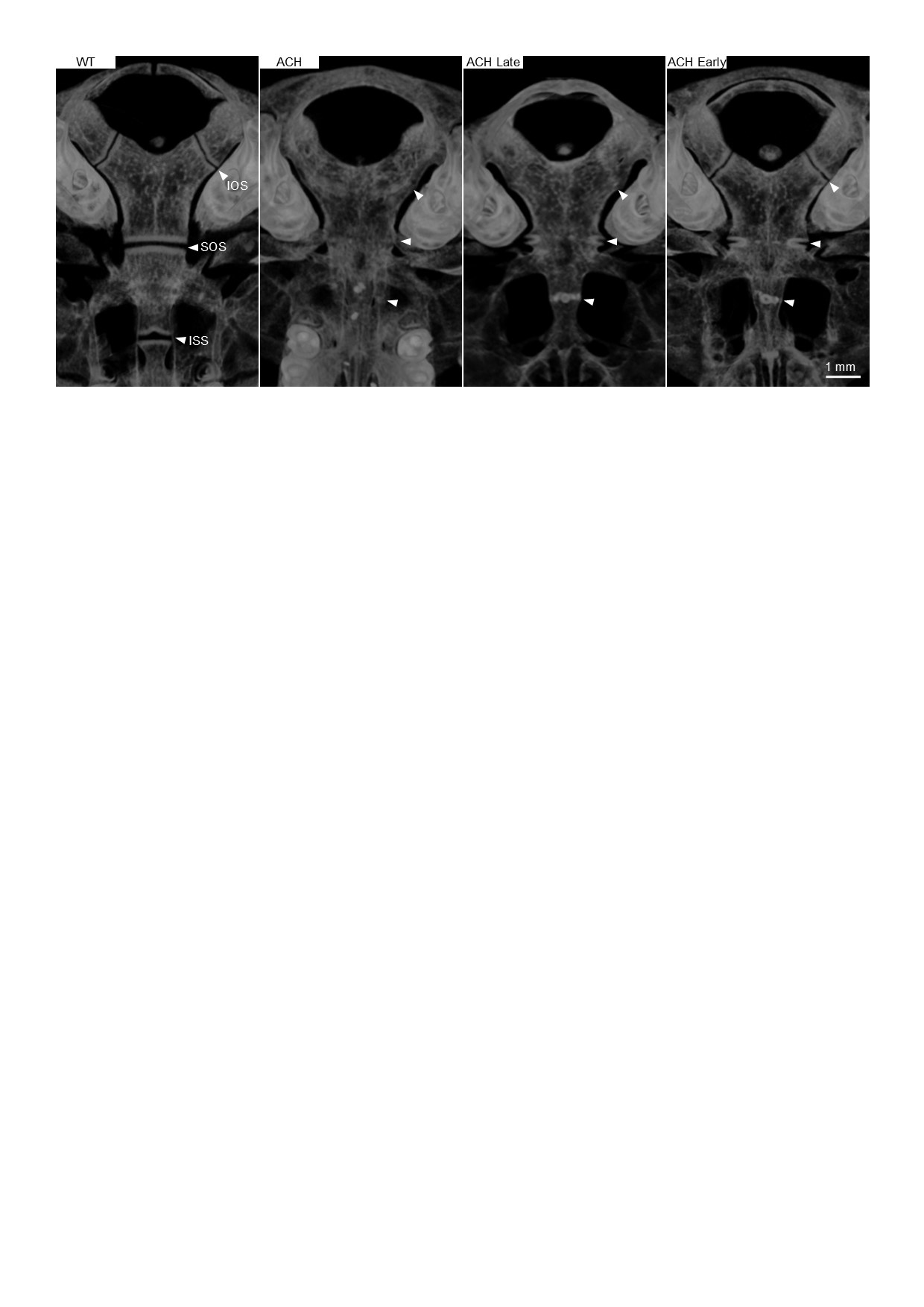


Representative images of reconstructed microCT scans were used to measure the degree of synchondroses (arrow) closure at P14 in wildtype (WT) and ACH mice treated with early (P1-14) and late (P4-P14) infigratinib protocols.

**Figure S7** Insufficient prenatal growth of cranial base in achondroplasia


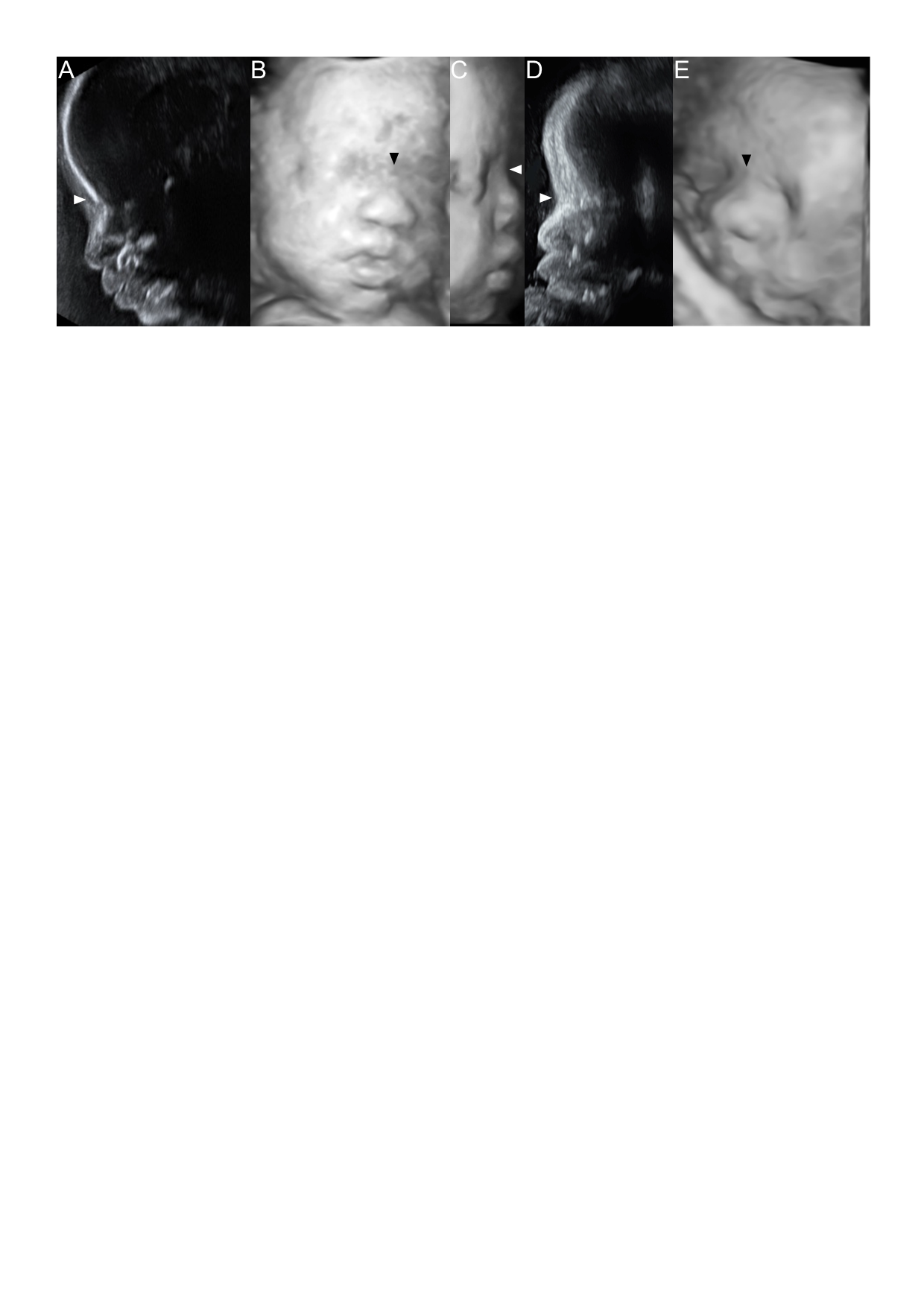


Profile and facies of two fetuses with achondroplasia. (**A**-**C**) 29-week gestational age fetus. Arrows in (**A**) and (**C**) point to depressed nasal bridge with frontal bossing and rounded nasal tip, on facial profile by 2D and 3D ultrasound. (**B**) 3D ultrasound of the fetal facies, black arrow points to depressed nasal bridge. (**D**, **E**) 32-week fetus. (**D**) Arrow points to frontal bossing, depressed nasal bridge and rounded nasal tip by 2D ultrasound. (**E**) Arrow points to depressed nasal bridge seen by 3D ultrasound.
