# Supplementary material for: Achondroplasia: aligning mouse model with human clinical studies shows crucial importance of immediate postnatal start of the therapy
Source: J Bone Miner Res. 2024 Oct 18;39(12):1783–92. doi: 10.1093/jbmr/zjae173 (PMC11638852; doi:10.1093/jbmr/zjae173)
Supplement: Supplementary_Table_1_zjae173 [file supplementary_table_1_zjae173.docx]

**Table S1** Number of animals used in experiments

|  | *WT* | | | *ACH* | | |
| --- | --- | --- | --- | --- | --- | --- |
| *Measurement (figure)* | *Control* | *Late* | *Early* | *Control* | *Late* | *Early* |
| Femur length (1C) | 20 | 7 | 8 | 26 | 9 | 11 |
| Cortical bone density (1D) | 20 | 7 | 8 | 26 | 9 | 11 |
| Fronto-basal angle (1H) | 20 | 7 | 8 | 26 | 9 | 11 |
| Foramen magnum area (1I) | 20 | 7 | 8 | 26 | 9 | 11 |
| Skull length (5E) | 20 | 7 | 8 | 26 | 9 | 10 |
| Body length (S2A) | 20 | 7 | 8 | 26 | 9 | 10 |
| L4-L6 (S2B) | 20 | 7 | 8 | 26 | 9 | 10 |
| Tail length (S2C) | 15 | 7 | 8 | 21 | 9 | 10 |
| Tibia length (S3) | 20 | 7 | 8 | 26 | 9 | 10 |
| Synch. open area at P8 (3B, 4C, 5B) | 11 | 11 | 12 | 7 | 14 | 14 |
| Synch. coverage at P14 (3F, 4H, 5G) | 20 | - | - | 26 | 9 | 11 |

A

|  |  | *Days after birth* | | | | | |
| --- | --- | --- | --- | --- | --- | --- | --- |
| *Measurement (figure)* | *Genotype* | *P2* | *P4* | *P6* | *P8* | *P14* | *P21* |
| Synch. open area (2B) | WT | 3 | 3 | 6 | 11 | 10 | 10 |
| Synch. open area (2B) | ACH | 6 | 8 | 4 | 7 | 10 | 10 |

B

|  |  | *Infigratinib (mg/kg)* | | | |
| --- | --- | --- | --- | --- | --- |
| *Measurement (figure)* | *Genotype* | *Control* | *0.5* | *1* | *1.5* |
| Body length (S1B) | WT | 20 | 7 | 2 | 8 |
| Weight (S1B) | WT | 20 | 7 | 2 | 8 |
| Skull length (S1B) | WT | 20 | 7 | 2 | 8 |
| Skull length:width (S1B) | WT | 20 | 7 | 2 | 8 |
| Fronto-basal angle (S1B) | ACH | 26 | 5 | 6 | 10 |
| Foramen magnum area (S1B) | ACH | 26 | 5 | 6 | 10 |
| Femur length (S1B) | ACH | 26 | 5 | 6 | 10 |
| Tibia length (S1B) | ACH | 26 | 5 | 6 | 10 |

C

(**A**) Number of animals used in experiments with 1.5 mg/kg infigratinib. (**B**) Number of animals used to document synchondroses development. (**C**) Number of animals in infigratinib toxicity and efficacy experiments. WT, wildtype; Late, late treatment protocol; Early, early treatment protocol.
